# EMPOWERMENT AMONG FORMAL CAREGIVERS WORKING WITH PERSONS WITH DEMENTIA IN HOME CARE

**DOI:** 10.1093/geroni/igz038.1742

**Published:** 2019-11-08

**Authors:** Heather A McIlveen, Marie Y Savundranayagam, J B Orange, Marita Kloseck

**Affiliations:** Western University, London, Ontario, Canada

## Abstract

There is significant literature on workplace empowerment that focuses on individuals in positions of power rather than those who lack it. However, there is limited research on empowerment of home care workers, such as personal support workers (PSW) who work in dementia care. Empowerment is an active process based on a multifaceted model consisting of four components: meaning, self-determination, impact and competence. This study explored the roles of education and employer support in empowering PSWs to care for persons with dementia who live at home. Empowerment was investigated using semi-structured interviews with PSWs (N=15). A phenomenological approach was to understand the lived experiences of home-care based PSWs who work with persons with dementia. Components of empowerment were reflected through five emerging themes: “providing best care”, “autonomy”, “employer support”, “career long learning”, and “experiential learning”. The theme “providing best care possible” support the component of meaning, which included the motivation for training among PSWs and their value of aging in place. The theme “autonomy” supported the component of self-determination, which focused on PSW workload and feelings regarding their control working in home care versus long term care. The theme “employer support” supported the component impact, which included both PSW compensation and their perceived lack of emotional support. Finally, the themes “career-long learning” and “experiential learning”, were linked with impact and competence components, respectively. Overall, these findings support relationships between education and employer support in empowering PSWs who care for persons with dementia who live at home.

